# Induction of IL-10 cytokine and the suppression of T cell proliferation by specific peptides from red cell band 3 and in vivo effects of these peptides on autoimmune hemolytic anemia in NZB mice

**DOI:** 10.1007/s13317-017-0095-4

**Published:** 2017-04-28

**Authors:** Abdel-Rahman Youssef, Christopher J. Elson

**Affiliations:** 1 School of Cellular and Molecular Medicine, University of Bristol, Biomedical Sciences Building, Bristol, BS8 1TD UK; 2 Department of Microbiology and Immunology, Faculty of Medicine, Suez Canal University, Ismailia, Egypt

**Keywords:** Autoimmune hemolytic anemia, IL-10, CD4 T cells, Band 3 peptides

## Abstract

**Purpose:**

The anion channel protein band 3 is the main target of the pathogenic red blood cells (RBC) autoantibodies in New Zealand black (NZB) mice. CD4 T cells from NZB mice with autoimmune hemolytic anemia respond to band 3. Previously, we have shown that IL-10 and peptides containing a dominant T cell epitope from red cell band 3 modulate autoimmune hemolytic anemia in NZB mice. Because of the immunoregulatory role of IL-10 in autoimmune diseases, we aim to identify individual band 3 peptides that induce high IL-10 production and simultaneously suppress CD4 T cell proliferation and to investigate the effect intranasal administration of IL-10 producing band 3 peptides on autoantibody responses of NZB mice.

**Methods:**

Splenic CD4 T cells of NZB mice were isolated and stimulated by co-culture of T cells with individual band 3 peptides. IL-10 production was measured by enzyme-linked immunosorbent assay and proliferative response of CD4 T cells was estimated by incorporation of [^3^H] thymidine assay. NZB mice were given either PBS, or peptides 25 (241–251) and 29 (282–296) or both peptides intranasally on three occasions at 2-day intervals. The mice were bled at 6, 10 and 18 weeks after peptide inhalation, and the number of RBC auto-antibodies was measured by DELAT and hematocrit values were assessed.

**Results:**

Peptides 25 (241–251) and 29 (282–296) induced the highest IL-10 production by CD4 T cells. These peptides also inhibited the peak T cell proliferative response. 6 and 10 weeks after peptide inhalation, the total IgG, IgG1 and IgG2a in mice treated with both peptides 241–251 and 282–296 were significantly higher than control (*P* < 0.05). However, no significant difference in the mean hematocrit between of the peptide-treated mice and the control group was found.

**Conclusions:**

Although band 3 peptides 241–251 and 282–296 induced to the highest IL-10 production by CD4 T cells in vitro but fail to reverse the RBC autoantibody response in vivo. Modifications to improve solubility these peptides might help to modulate the immune response toward a T helper-2 profile and decrease the severity of anemia.

## Introduction

There is a pressing need to develop specific immunotherapies for autoimmune diseases, and considerable attention is focused on the potential beneficial effects of peptides recognized by auto-reactive CD4 T cells. However, before such treatments can become a reality for human disease, it is important to understand the mechanisms underlying the action of these peptides, particularly in antibody-mediated conditions, where their effects are less well understood. Spontaneous autoimmune hemolytic anemia (AIHA) in the New Zealand Black (NZB) mice is a classic example of autoimmune antibody-mediated disease. NZB mice produce red blood cell (RBC)-bound IgG autoantibodies from as early as 6 weeks of age [1, 2] and develop signs of anemia some 5 months later [3]. The major target of the pathogenic RBC autoantibodies is the anion channel protein band 3 [4], and CD4 T cells from NZB mice respond to band 3 [1]. The optimal generation of these autoantibodies is CD4 T cell-dependent since production of the autoantibody is retarded in NZB mice treated with anti-CD4 antibodies [5] and in CD4-deficient mice [6]. More recently, it has been demonstrated that band 3 peptide 861–874 is the predominant sequence recognized by NZB T cells in vitro [7]. Mapping studies revealed that the peptide invariably induces strong proliferation of NZB CD4 T cells in vitro, and is the only sequence to stimulate responses by T cells from NZB mice prior to the onset of disease [8]. In addition, NZB mice given this (relatively insoluble) peptide develop a more severe disease. By contrast, the severity of anemia is reduced in NZB mice given a soluble analog of the peptide [9], showing that this disease is susceptible to peptide therapy.

IL-10 is anti-inflammatory cytokine with potent properties that maintains normal tissue homeostasis. Impairment of IL-10 is associated with high risk for development of many autoimmune diseases [10]. On the other hand, injecting NZB mice with plasmids encoding IL-10 (pIL-10) delayed the development of anemia [11]. Regulatory T cells control the activity of self-reactive cells in their lymphoid organs [12]. Previous studies have shown that IL-10 producing regulatory T cells were able to prevent or suppress autoimmune diseases. Myelin basic protein (MBP) peptide 1–9 was recognized by auto-reactive CD4 T cells and prevented the induction of an autoimmune disease. Intranasal administration of this peptide prevents the induction of experimental autoimmune encephalomyelitis (EAE) in mice by induction of regulatory T cells which produced IL-10 [13, 14]. In addition, IL-10-producing B cells (regulatory B10 cells) attenuate autoimmune, inflammatory reactions, through IL-10 production [15] and the administration of IL-10 can suppress collagen-induced arthritis [16], as well as the diabetes of non-obese diabetic (NOD) mice [17].

In human autoimmune hemolytic anemia, the Rhesus (Rh) complex carries autoreactive helper determinants since Rhesus peptide-specific T cells that have been activated in vivo, can be detected in the peripheral blood of all patients with anti-Rh autoantibodies [18]. Epitope mapping studies in AIHA patients identified peptides from the sequence of the RhD protein that induced IL-10 and suppressed T cell proliferation, and this inhibition was mediated by IL-10 [19]. Thus, the aim of the current work was to investigate the role of band 3 peptides in the induction of IL-10 and the suppression of T cell proliferation in the NZB mice model and to determine whether intranasal administration of the IL-10 producing band 3 peptides to NZB mice would modulate the response of the corresponding T cells and RBC autoantibody production and ameliorate NZB AIHA.

## Materials and methods

### Mice

NZB (H-2^d^) mice were maintained under specific pathogen-free conditions in the animal facilities at the University of Bristol. All animal experiments complied with UK Home office regulations, and the ‘Principles of laboratory animal care’ were followed throughout.

### Peptides

Band 3 peptides [20] were synthesized in the Department of Biochemistry, University of Bristol, UK by a solid-phase synthetic method using fluorenylmethoxycarbonyl-polyamide chemistry [21]. Individual band 3 peptides were suspended in phosphate buffered saline (PBS) containing 5% dimethyl sulphoxide (DMSO; Sigma) at 2 mg/ml.

### Administration of peptides

Groups of 6-week-old NZB mice were given 25 µL of band 3 peptides 25 (241–251) and 29 (282–296) or both in PBS (4 mg/mL) intranasally on three occasions (300 µg in total) at 2-day intervals. The control group received PBS.

### Stimulation of T cell

Spleens were harvested from mice and purified CD4 T cells (>95% CD4 as determined by flow cytometry analysis) were obtained by positive selection using magnetic beads coated with anti-CD4 mAb (Miltenyi Biotec, Bergisch Gladbach, Germany) according to the manufacturer’s instructions.

The CD4 cells were resuspended in alpha modification of Eagle’s medium supplemented with 20 mM HEPES buffer, 100 U/ml benzyl penicillin, 100 μg/ml streptomycin sulfate, 4 mM l-glutamine and 50 μM 2-mercaptoethanol. Antigen-presenting cells (APCs) were produced by irradiation of the non-CD4+ cells with 1500 rads (caesium source) to prevent cell division. APCs were mixed with CD4+ cells to reach a final concentration of 0.6 × 10^6^ cells/ml for APCs and 1.25 × 10^6^ cells/ml for CD4+ cells. 1% mouse serum was added as a protein source. Band 3 peptides were added at a final concentration of 20 or 200 μg/ml and the cells were incubated in a humidified atmosphere of 5% CO_2_ and 95% air at 37 °C. Dominant band 3 peptide 861–874 and its soluble analog as well as concanavalin A (ConA) were used as positive controls.

### Cell proliferation assay

Cellular proliferation was estimated by incorporation of [^3^H] thymidine. Cell proliferation was measured over four days from day 3 to day 6 of culture. 100 μl samples of each CD4 T cell culture were added in triplicate to a 96-well plate and incubated for 6 h with 1 μCi per well of [^3^H] thymidine. The cells were incubated in a humidified atmosphere of 5% CO_2_ and 95% air at 37 °C for 6 h. Cells were harvested on filter paper, and incorporation of tritiated thymidine was measured using a 1450 Microbeta Plus scintillation counter (LKB Wallac, Turku, Finland) in the department of Cellular and Molecular Medicine, Bristol University, UK.

### ELISA

Production of IL-10 was assessed on day 4/5 of cell culture by CelELISA for mouse IL-10 (Biosource International, Nivelles, Belgium) as described previously [20]. Briefly, cell samples, set up in triplicate, were cultured overnight in a plate coated with rat anti-mouse IL-10 at 37 °C in a humidified atmosphere of 5% CO_2_. Post incubation, IL-10 was detected with biotinylated rat anti-mouse IL-10. The optical densities of plate wells were measured at wavelength 450 nm and IL-10 levels were calculated by regression analysis against standard curves produced with the appropriate recombinant cytokine.

### Measurement of IgG subclasses bound to RBC

The levels of IgG molecules bound to the surface of RBC were measured by direct enzyme-linked anti-globulin test (DELAT). Briefly, a 2% suspension of fixed RBC were incubated with subclass-specific sheep anti-mouse IgG antibody (The Binding Site, Birmingham, United Kingdom) for 1 h at 37 °C. After, the RBC were incubated with alkaline phosphatase–conjugated donkey antisheep antibody (Sigma) then washed and allowed to react with phosphatase substrate solution (p-nitrophenyl phosphate; Sigma) for 1 h at 37 °C. The optical density (OD) measured at 405 nm (Titertek Multiscan II; Labsystems, Hants, United Kingdom). The number of molecules of each murine IgG subclass bound to the RBC was calculated by interpolation from a standard curve generated by tanning normal RBC with a known concentration of each purified IgG subclass (Sigma).

## Results

### CD4 T cells production of IL-10 by after stimulation by band 3 peptides

**Fig. 1:**
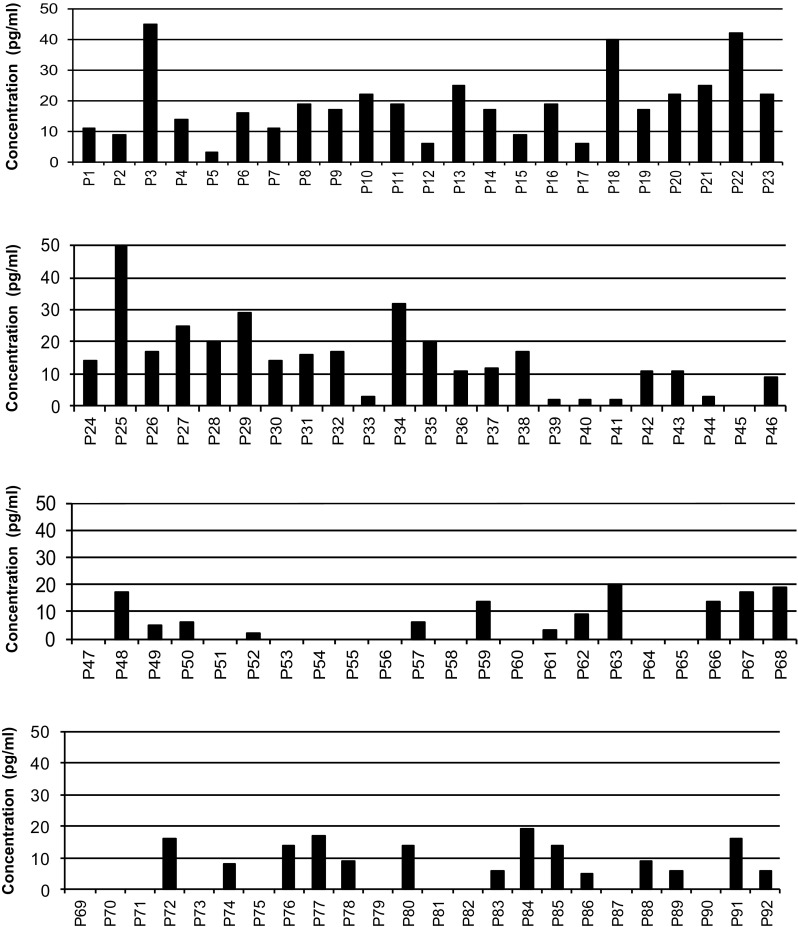
Production of IL-10 by CD4 T cells after band 3 peptide stimulation at a dose of 20 μg/ml. Production of IL-10 was assessed on day 4/5 of cell culture by CelELISA. The *X* axis represents the individual 92 peptides of band 3, while the *Y* axis shows the IL-10 concentration. IL-10 value without peptide was deducted from the test value. The data shown are means of duplicate samples representative of three independent experiments

**Fig. 2:**
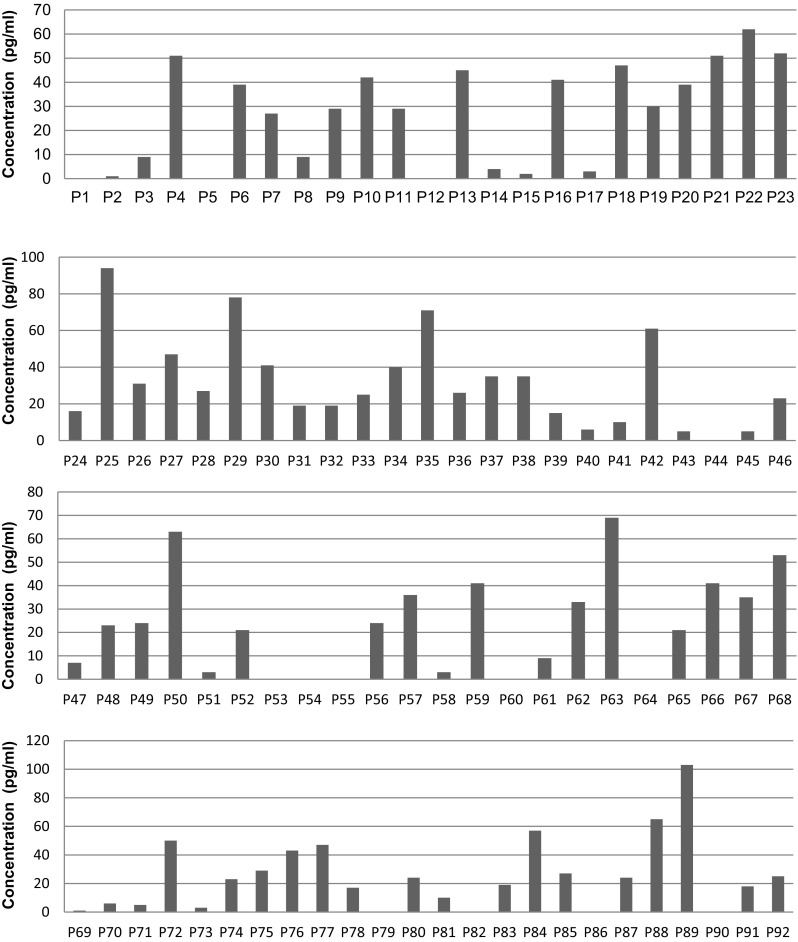
Production of IL-10 by CD4 T cells induced by 92 peptides of band 3 at a dose of 200 μg/ml. Production of IL-10 was assessed on day 4/5 of cell culture by CelELISA. The *X* axis represents the individual 92 peptides of band 3, while the *Y* axis shows the IL-10 concentration. IL-10 value without peptide was deducted from the test value. The data shown are means of duplicate samples representative of three independent experiments

We first examined the IL-10 production by CD4 T cells in the presence of the individual 92 peptides at two concentrations 20 μg/ml and 200 μg/ml. Generally, peptides were able to stimulate higher IL-10 production at a concentration of 200 μg/ml than at 20 μg/ml. At the concentration of 20 μg/ml, (Fig. 1) only four peptides (3, 18, 22, 25) were able to stimulate the highest IL-10 production. However, when the different 92 peptides were used at concentration of 200 μg/ml (Fig. 2) fourteen peptides at the tested dose were able to stimulate higher IL-10 production. These peptides include peptides 4, 21, 22, 23, 25, 29, 35, 42, 50, 63, 68, 84, 88 and 89. The sequence and positions of these peptides are shown in Table 1 [22].

**Table 1:** The sequences and positions of selected peptides of band 3 proteins

Peptide no.	Amino acid sequence	Position
4	YRDLTIPVTEMQDPE	41–55
13	SLLELQKVFSKGTFL	120–134
18	LLLKRSHAEDLGNLE	171–185
22	ETQLYCGQAEGGSEG	210–224
25	LVLVGRANFLEKPVL	241–251
29	LGPEAPHVDYTQLGR	282–296
34	SLVLPPTDAPSEKAL	331–345
35	PSEKALLNLVPVQKE	340–354
42	PYYLSDITDALSPQV	410–424
50	EEAFFSFCESNNLEY	491–505
63	RVIGDFGVPISILIM	621–635
68.	LGLYRLFPTWMMFASV	671–686
88	STPASLA LPFVLILT	870–884
89	VLILTVPLRRLILPLIFR	880–897
Dominant peptide	CLAVLWVVKSTPAS	861–874

### Effect of IL-10 producing peptides on CD4 T cell proliferation

**Fig. 3:**
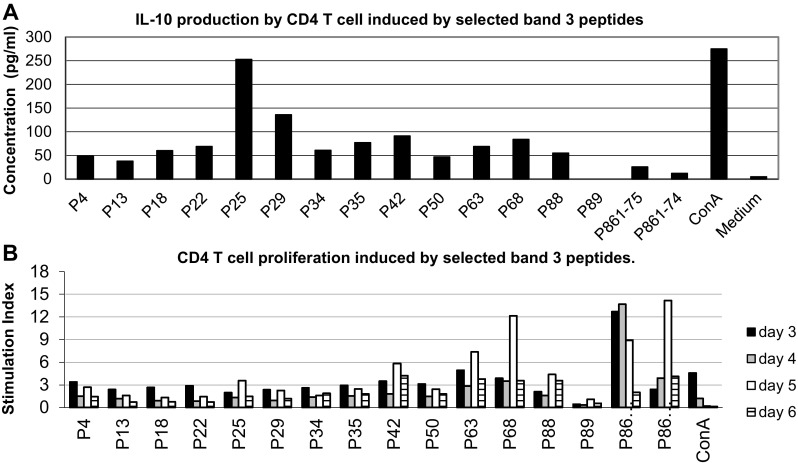
IL-10 production and CD4 T cell proliferation elicited by selected band 3 peptides. The peptides were selected according to their ability to induce IL-10 production. CD4 T cell were stimulated with selected peptides at a dose of 200 μg/ml and IL-10 production was calculated by CelELISA (**a**) and stimulation index of proliferative response was measured by incorporation of [^3^H] thymidine (**b**). T cell proliferation as assessed from day 3 to day 6 after co-culture with peptides. The proliferative response is expressed as a stimulation index (SI) calculated by dividing the mean cpm of peptide-stimulated wells by the mean cpm of non-stimulated wells. The data shown are means of duplicate samples representative of three independent experiments

Since IL-10 is considered to be a regulatory cytokine, the ability of these peptides to suppress the proliferative response of CD4 T cell was examined parallel to the IL-10 production. The proliferative response is expressed as a stimulation index (SI) which is calculated by dividing the mean counts per minute (cpm) of peptide-stimulated wells by the mean cpm of non-stimulated wells. As can be seen in Fig. 3, peptides 25 (241–251) and 29 (282–296) induced the highest IL-10 production by CD4 T cells and inhibited T cell proliferation (SI less than 2.5) compared to the proliferative response of dominant band 3 peptide 861–874 and its soluble analog with a stimulation index more than 12.

### Effect of mucosal administration of band 3 peptides 241–251 and 282–296, on NZB mice autoantibody responses

**Fig. 4:**
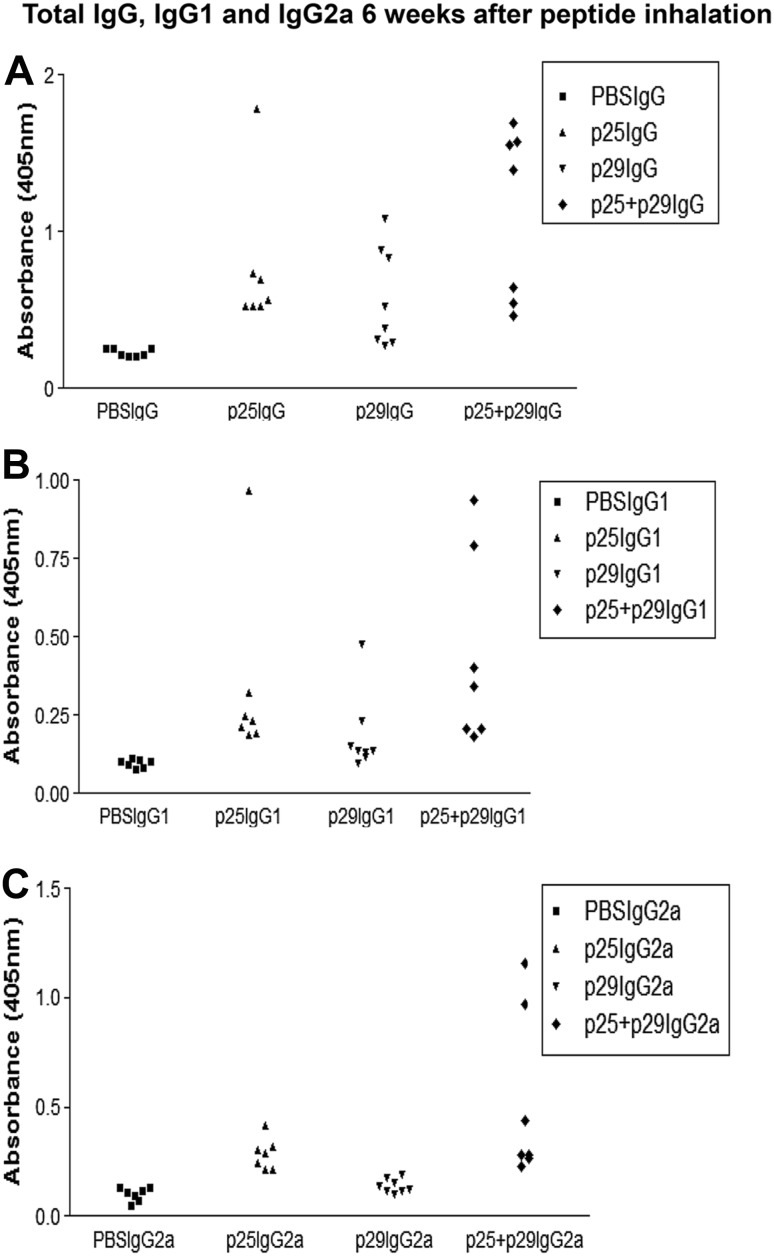
RBC autoantibody responses 6 weeks after peptide inhalation. Comparison of total IgG (**a**), IgG1 (**b**) and IgG2a (**c**) responses of individual NZB mice 6 weeks after they had inhaled PBS or peptide 25 (241–251) or peptide 29 (282–296) or both (p25 + p29). Total IgG was significantly higher in p25 and p25 + p29 groups (*P* < 0.05) and both IgG1 and IgG2a were significantly higher in p25 + p29 group in comparison to control (*P* < 0.05) (One-way ANOVA)

**Fig. 5:**
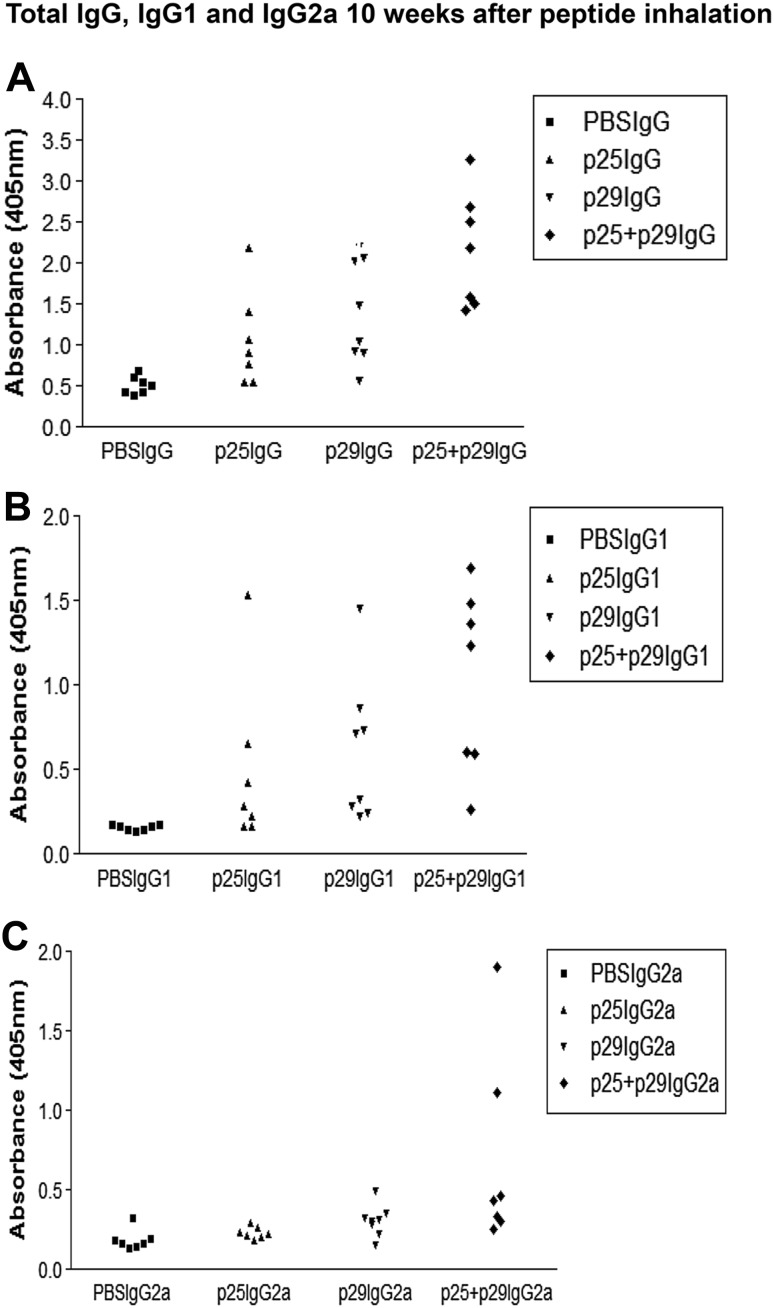
Total IgG, IgG1 and IgG2a 10 weeks after peptide inhalation 10 weeks after peptide inhalation. Comparison of total IgG (**a**), IgG1 (**b**) and IgG2a (**c**) responses of individual NZB mice 10 weeks after they had inhaled PBS (*n* = 7) or p25 or p29 or p25 + p29. Total IgG was significantly higher in p25 (*P* < 0.05) and p25 + p29 (*P* < 0.01) groups and both IgG1 and IgG2a were significantly higher in p25 + p29 group in comparison to control (*P* < 0.01) (One-way ANOVA)

An experiment was set up to determine if peptide inhalation affected the level of each IgG isotype of the RBC-bound autoantibodies. Groups of NZB mice that had inhaled PBS, band 3 peptides 25 (241–251) and 29 (282–296) or both peptides (pMix). The mice were bled at 6 and 10 weeks after inhalation, and the number of antibody molecules of each isotype present on their RBC was measured by DELAT. When autoantibody responses was analyzed after 6 weeks (Fig. 4) and 10 weeks (Fig. 5) following peptide inhalation, the total IgG was significantly higher in p25 and p25 + p29 groups (*P* < 0.05) and both IgG1 and IgG2a were significantly higher in p25 + p29 group (*P* < 0.05) compared to control (One-way ANOVA).

### Effect of mucosal administration of band 3 peptides 241–251 and 282–296 on NZB anemia

**Fig. 6:**
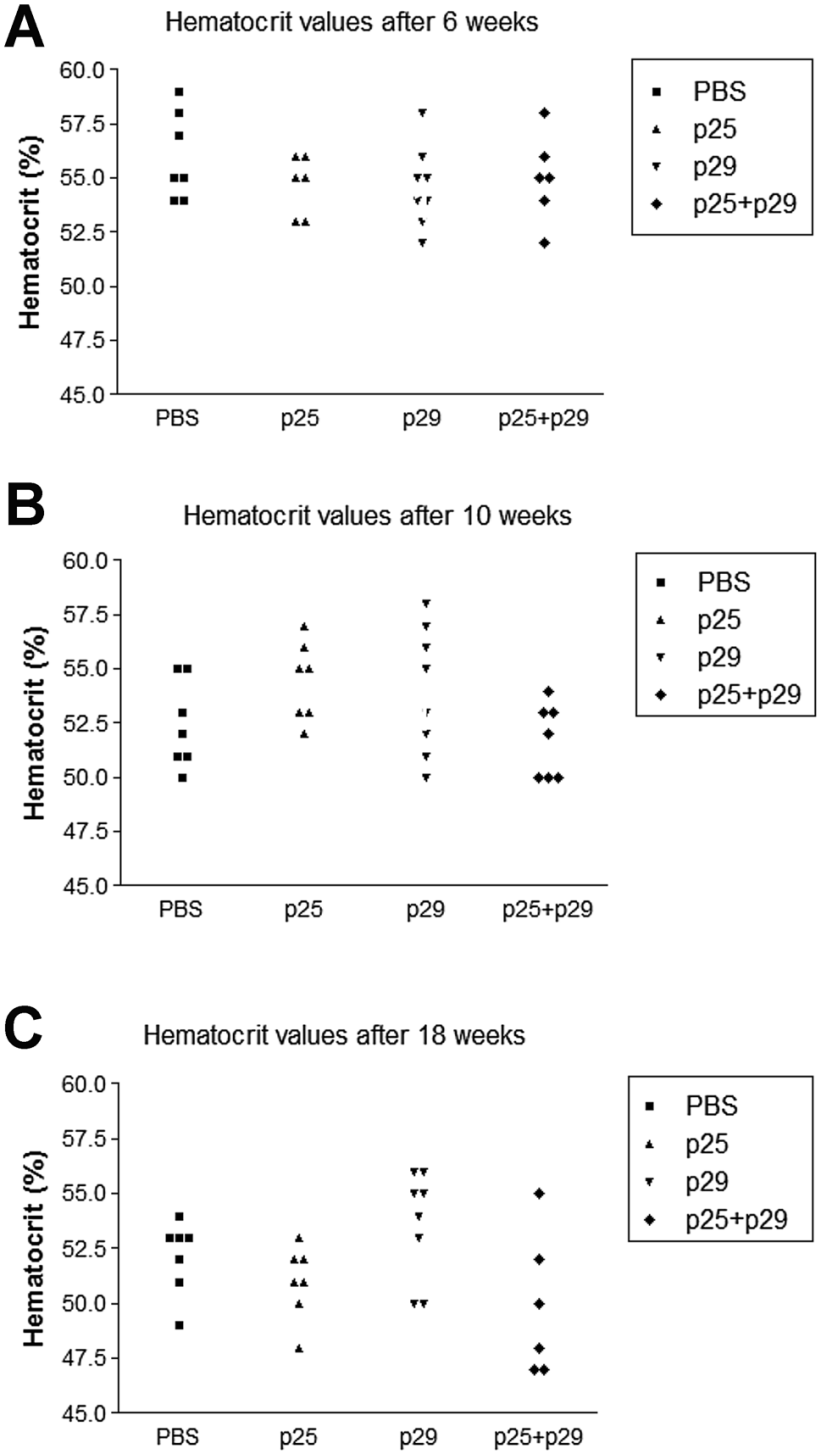
Comparison of hematocrit values of individual NZB mice. The mice had inhaled or peptide 25 (241–251) or peptide 29 (282–296) or both (p25 + p29) and hematocrit values were measured after 6 weeks (**a**) 10 weeks (**b**) and 18 weeks (**c**). No significant difference between the groups (*P* > 0.05) (One-way ANOVA) was found at any time point

To test if mucosal administration of band 3 peptides 241–251 and 282–296 affect the rate of hemolysis, groups of NZB mice were given peptides or PBS intranasally and the development of anemia was compared. As can be seen in Fig. 6, after 6, 10 and 18 weeks of treatment, no significant difference in the mean hematocrit between of the peptide-treated mice and the control group (*P* > 0.05) (One-way ANOVA).

## Discussion

NZB mouse model has been used extensively for the analysis of spontaneous AIHA [1, 2]. In this study we mapped band 3 peptides that are able to produce IL-10 and we further investigated the effect of selected IL-10-producing peptides on CD4 T cell proliferation. We found that peptides 25 (241–251) and 29 (282–296) induced the most IL-10 and suppressed the peak proliferative response of dominant band 3 peptide 861–874 and its soluble analog. These data suggest that peptides 25 (241–251) and 29 (282–296) have the potential to be tested as a therapeutic reagent for AIHA studies.

A number of studies have shown that peptide therapy induced IL-10-dependent immunological tolerance. In NOD mice, peptide from heat-shock protein, p277, arrested beta-cell destruction, maintained insulin production [23] and induced Th2 cytokines (IL-4 and IL-10) [24]. In human, cat allergen peptide therapy showed decreased Th1 and Th2 with increased IL-10 [25] and grass pollen immunotherapy resulted in higher IL-10 production compared to atopic control subjects [26]. Peptides derived from the antigens associated with allergy and autoimmune disease may be used as vaccines to treat these disorders [27]. In addition, administration of the plasmids encoding IL-10 (pIL-10) delayed the development of anemia in NZB mice as judged by increased haematocrit values compared with controls [11].

How might IL-10-producing peptide influence tolerance in NZB model? IL-10 may play a role in both the induction of regulatory cells and their effector function. IL-10 induced differentiation of CD4 T cells into type 1 regulatory T (Tr1) cells and prevented colitis induced in severe combined immunodeficient (SCID) mice by pathogenic CD4 + CD45RB (high) splenic T cells. [28]. Additionally, transfer of Tr1 T cell clones inhibited Ag-specific serum IgE responses through IL-10 secretion in immediate hypersensitivity murine model [29]. Moreover, the MBP peptide 1–9 induced regulatory T cells of the Tr1 type in vivo, and neutralization of IL-10 prevented the peptide MBP from protecting against experimental autoimmune encephalomyelitis [14]. Furthermore, IL-10 was shown to be essential for the protection of C57BL/6 mice from experimental autoimmune encephalomyelitis after administration of the soluble peptide 35–55 from myelin oligodendrocyte glycoprotein [30].

It follows that, at least in some systems; peptides recognized by auto-reactive CD4 T cells exert their therapeutic effect by an IL-10-dependent mechanism. Such studies, together with the fact that administration of IL-10 can suppress collagen-induced arthritis [16], the diabetes of NOD [17] mice as well as experimental autoimmune encephalomyelitis [31], support the potential therapeutic use of band 3 peptides 241–251 and 282–296 in autoimmune hemolytic anemia.

It is known that Th1 cytokine interferon-gamma and Th2 cytokine IL-4 stimulate isotype switching to IgG2a and IgG1, respectively [32, 33]. Autoantibody subclasses IgG2a and IgG1 immunoglobulin isotypes were investigated as indicative of a Th1 and Th2 type responses, respectively [34].

Previously we have demonstrated that development of RBC autoantibodies in NZB mice is associated with Thl cytokine-dominated responses [2]. In vivo experiments were planned to examine the effect of inhaling the band 3 peptides that induced IL-10 on NZB anemia. It was expected this treatment would deviate the immune response toward Th2 cytokines and reduce hemolysis. However in this study we demonstrated a significant enhancement of both Th1 and Th2-related IgG subclasses in response to inhaled peptides and no improvement in anemia after 18 weeks of treatment. Our findings are consistent with other studies that have described enhancement of both Th1 and Th2 in response to subcutaneous immunization of ovalbumin dissolved in saline containing Chitosan nanoparticles in mice [35] and to inhaled allergic in atopic children [36]. The results of in our in vivo experiment may be due to the fact that in NZB disease T cells primed to inhaled peptides were already generated by the time therapy was initiated and is likely to reflect the expansion of CD45RO memory cells [37]. In addition, solubility of peptides can be a determining factor as insolubility is often associated with immunogenicity rather than tolerogenicity. This was clear when inhalation of the insoluble peptide 861–874 primed T cells for both peptide 861–874 and band 3 responses whereas inhalation of a soluble analog (Glu861, Lys875) of peptide 861–874 deviated the autoimmune response toward a Th2 profile [9].

In conclusion, peptides 241–251 and 282–296 stimulated the highest IL-10 production by CD4 T cells and inhibited the peak T cell proliferative response but failed to induce tolerance in vivo and accelerated the priming of band 3-reactive T cells. Designing a soluble analog of band 3 peptides 241–251 and 282–296 should be considered before intranasal immunization.

## Abbreviations

RBC: Red blood cells
NZB: New Zealand black
AIHA: Autoimmune hemolytic anemia
ELISA: Enzyme-linked immunosorbent assay
pIL-10: Plasmids encoding IL-10
NOD: Non-obese diabetic
MBP: Myelin basic protein
EAE: Experimental autoimmune encephalomyelitis
PBS: Phosphate buffered saline
DMSO: Dimethyl sulphoxide
APCs: Antigen-presenting cells
ConA: Concanavalin A
Tr1: Type 1 regulatory T
